# Repairing the N-vacancy in an InN monolayer using NO molecules: a first-principles study

**DOI:** 10.1039/c9na00041k

**Published:** 2019-03-29

**Authors:** Hao Cui, Dachang Chen, Chao Yan, Ying Zhang, Xiaoxing Zhang

**Affiliations:** State Key Laboratory of Power Transmission Equipment & System Security and New Technology, Chongqing University Chongqing 400044 China xiaoxing.zhang@outlook.com; School of Electrical and Computer Engineering, Georgia Institute of Technology Atlanta 30332 GA USA yzhang@gatech.edu; School of Electrical Engineering, Wuhan University Wuhan 430072 China; Key Laboratory of Education Ministry for Modern Design and Rotor-Bearing System, Xi'an Jiaotong University Xi'an 710049 China

## Abstract

The synthesis of a perfect InN monolayer is important to achieve desirable properties for the further investigation and application of InN monolayers. However, the inevitably existing defects, such as an N-vacancy, in the synthesized InN nanomaterials would significantly impair their geometric and electronic behaviors. In this study, we proposed to repair the N-vacancy in the InN monolayer using NO molecules through NO disproportionation, which was verified to be energetically favorable according to our first-principles calculations. The repaired InN monolayer was similar to the perfect counterpart in terms of the geometric and electronic aspects. In this study, a promising strategy is presented for repairing the N-vacancy in the InN monolayer to perfect its physicochemical properties effectively, which may also be used to repair N-vacancies in other materials.

## Abstract 2

The synthesis of a perfect InN monolayer is important to achieve desirable properties for the further investigation and application of InN monolayers.

## Graphical abstract

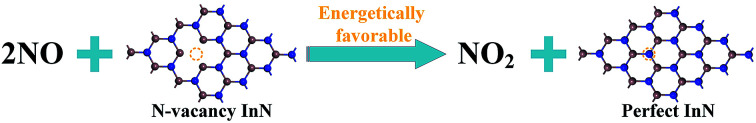

## 1 Introduction

Two-dimensional (2D) materials are always the focus of attention due to their unique electronic behavior, high charge-carrier mobility, large specific surface area and excellent optical property,^1,2^ which enable their applications in many fields. The first real 2D material with one-atom thickness is graphene; however, its gapless nature limits its application in logic and high-speed switching devices.^3^ Thus, other candidate materials with a graphene-like structure, tunable band gap and similar or even better properties for specific applications need to be explored.^4–7^

Ultrathin III–V compounds with graphene-like microscopic structures and direct-bandgaps have recently received significant attention.^8–13^ Among them, after the remarkable breakthrough in the synthesis of nano-scaled InN, InN monolayers have been widely studied for potential application as gas nanosensors^14–16^ and optical coatings.^17,18^ However, there are some inevitable defects in the synthesized InN nanomaterials; for example, the N vacancies have low formation energy and can become more stabilized upon the incorporation of In vacancies; this leads to the formation of vacancy-complexes.^19,20^ Hence, the existence of N vacancies is harmful to the chemical stability and electronic behavior of the InN monolayer. It has been reported that unlike the case of multilayer InN, the electron mobility of the InN monolayer can be impaired pronouncedly as the number of vacancies increases;^21^ this largely deteriorates its physicochemical behavior. In this regard, the possible N vacancies should be repaired after the synthesis of the InN monolayer to obtain a perfect configuration with high material quality.

A novel method to perfect the InN monolayer was introduced in this study, wherein gaseous NO was proposed as the N source through NO disproportionation, specified as NO(g) + NO(g) → N(s) + NO_2_(g). This is an intriguing approach as it does not require metal catalysts, and the N-defected InN (Vac-InN) monolayer thus obtained behaves as a catalytic support, which heals itself by the N atom produced from the reaction. In fact, the repairing of vacancy using gas molecules has been investigated in the last several years. Liu proposed to heal the C-vacancy in graphene by the CO molecule based on an electric field method.^22^ Divacancy in graphene or carbon nanotubes could also be healed using C_2_H_2_ or C_2_H_4_ molecules.^23,24^ In the C_3_N monolayer, the C or N vacancies were also repaired using CO or NO molecules.^25^ Moreover, the interaction between gas molecules used to repair the vacancy and the 2D nanomaterials could exert significant effects on the electronic behavior of the substrates, such as the case of MoS_2_ and WSe_2_ monolayers in which an effective p-doping was achieved after gas adsorption on the vacancy sites.^26,27^ In this study, our calculations indicated that the processes of repairing the N-vacancy InN monolayer using the NO molecules were energetically favorable due to the small energy barrier and large energy drop in each reaction. These results, in line with the previous reports, verified the feasibility and efficiency of repairing 2D nanomaterials by related gaseous molecules. Thus, we are hopeful that our study can provide some guidance to synthesize a perfect InN monolayer for application in many fields.

## 2 Computational details

The whole structural relaxation and electronic calculations were performed within the dispersion-corrected density functional theory (DFT) of DMol^3^ package.^28^ Perdew–Burke–Ernzerhof (PBE) function within the generalized gradient approximation (GGA)^29,30^ was adopted to describe the electron exchange-correlation interaction. The DFT-D2 method developed by Grimme was employed to better understand the Van der Waals force and long-range interactions.^31^ Double numerical plus polarization (DNP) was selected as the atomic orbital basis set,^32^ with global orbital cut-off radius of 5.0 Å and smearing of 0.005 Ha to ensure a high computational quality. The Monkhorst–Pack *k*-point mesh of 10 × 10 × 1 was determined for all super-cell geometry optimizations and electronic structure calculations. Complete linear synchronous transit (LST)/quadratic synchronous transit (QST) calculations were performed to locate transition states (TS).^33^

We established a 4 × 4 × 1 intrinsic InN monolayer supercell with a vacuum region of 15 Å to prevent the interaction between adjacent units. The lattice constant of the fully optimized InN monolayer was 3.62 Å, which is in agreement with other theoretical work (3.63 Å (ref. 34)). The adsorption energy (*E*_ad_) was calculated as: *E*_ad_ = *E*_Surf/NO_ − *E*_Surf_ − *E*_NO_, where *E*_Surf_ and *E*_Surf/NO_ represent a total energy of the analyzed monolayer before and after NO adsorption, and *E*_NO_ is the energy for isolated NO molecule. Hirshfeld method was considered to analyze the atomic and molecular charge behaviors.

## 3 Results and discussion

### 3.1 Analysis of the N-vacancy InN monolayer

**Fig. 1:**
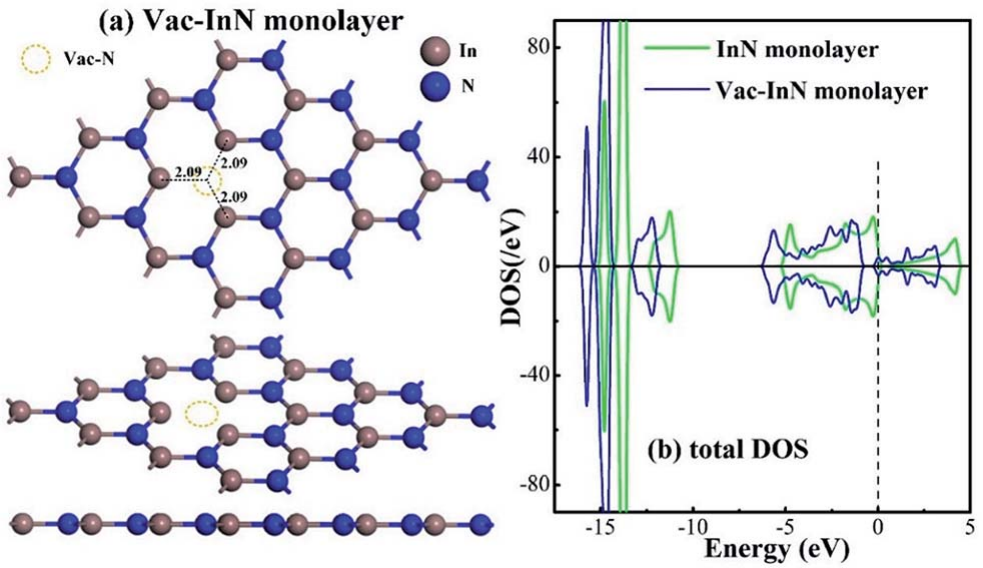
Geometric and electronic structure of the Vac-InN monolayer. (a) Configurations of the Vac-InN monolayer and (b) total DOS of pristine and N-defected InN monolayer. The black values are bond lengths (Å) and the dash line is the Fermi level.

Fig. 1 shows the geometric and electronic structures of the Vac-InN monolayer. It is found in Fig. 1(a) that the vacancy-In distances are 2.09 Å, which is equal to the length of In–N bond in the perfect InN monolayer. This reveals that the N defect causes slight deformation in the plane morphology of the InN monolayer. However, the electronic behavior of the InN monolayer undergoes significant changes within the N vacancy, as verified from the distributions of density of state (DOS) in Fig. 1(b). It can be seen that the DOS curves of the Vac-InN monolayer are left-shifted when compared with those of the intrinsic counterpart due to the strong donator-like states caused by the nonbonding electrons. It has been reported that these states can trap electrons and scatter other charge carriers in the InN monolayer,^35^ by which the carrier mobility would be reduced. Thus, repairing of the N vacancy is essential to guarantee the desirable property of the InN monolayer.

### 3.2 Adsorption of the NO molecule on the N-vacancy InN monolayer

Before conducting the repairing processes of N-vacancy in the InN monolayer, we investigated its adsorption behavior upon NO molecule, where two adsorption sites were considered, namely N-vacancy site and In-above site neighboring the N-vacancy. Moreover, in the abovementioned two sites, the NO molecule was placed at the N-end, O-end and molecule-parallel positions to the plane, in which the stability of each adsorbed configuration was determined by *E*_ad_.

**Fig. 2:**
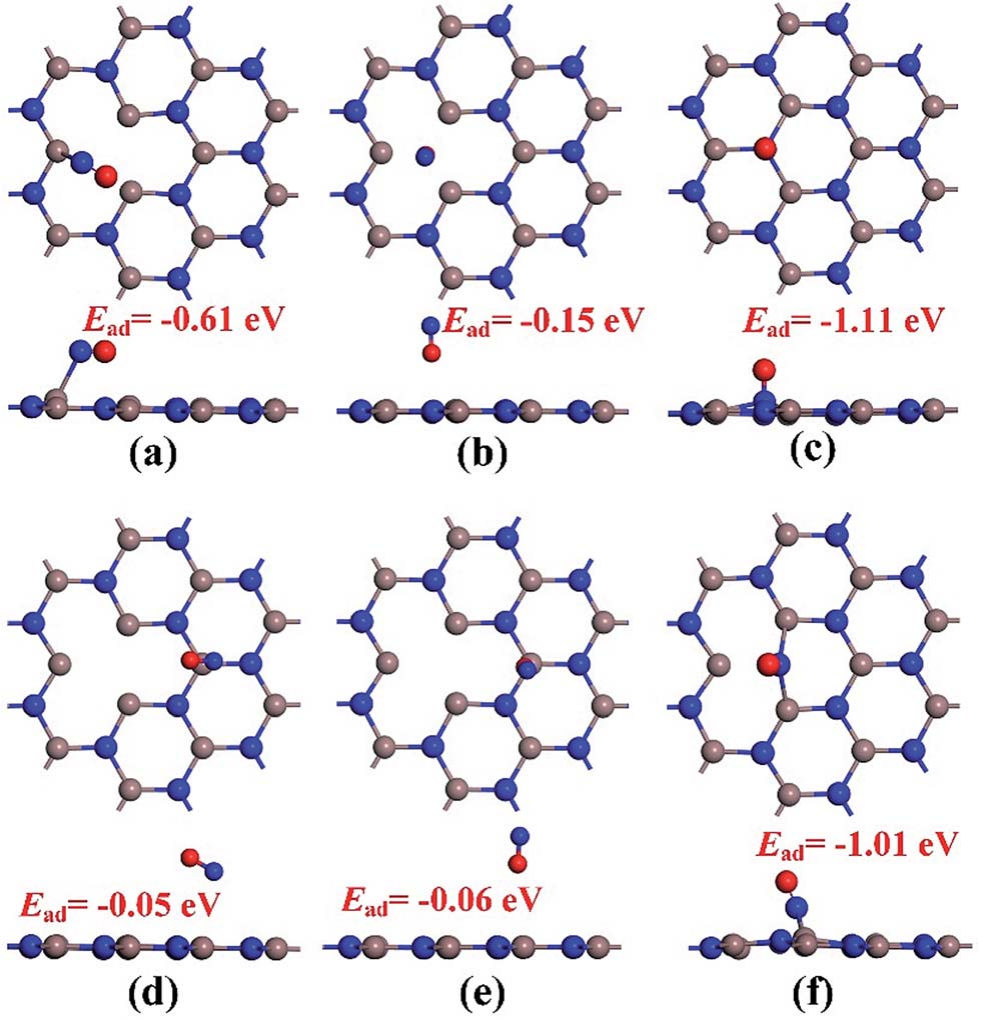
Configurations of NO adsorption on the N-vacancy site (a)–(c) and In-above site (d)–(f). (a) and (d) molecular parallel position; (b) and (e) O-end position; (c) and (f) N-end position.

The geometries of adsorption configurations after full optimization are shown in Fig. 2. It could be found that regardless of the N-vacancy site or the In-above site, the N-end position is the most energetically favorable structure with the calculated *E*_ad_ of −1.11 and −1.01 eV, respectively. Interestingly, at the In-above site with the N-end position, the NO molecule experiences a dramatic displacement and moves to the N-vacancy site with N atom captured by two dangling In atoms, similar to the structure of N-end position at the N-vacancy site. In practice, the configuration of the N-end position at the N-vacancy site is indeed most energetically favorable among all the structures. Thus, it was defined as the specific model for NO chemisorption, which has been analyzed in detail in the next section. Moreover, it shed light on the possibility of reparation of the N-vacancy InN monolayer using NO, where the adsorption of NO is the first step.

**Fig. 3:**
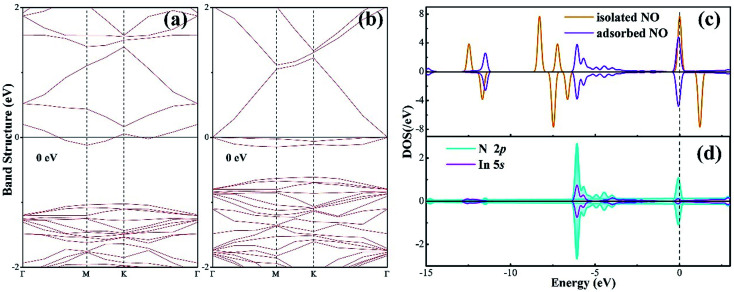
(a) BS of the pure N-vacancy InN monolayer; (b) BS of the NO adsorbed N-vacancy InN monolayer; (c) molecular DOS of NO and (d) atomic DOS.

To further understand the chemisorption of the NO molecule, the band structure (BS) and density of state (DOS) are plotted in Fig. 3. For better comparison, the band structure of the pure N-vacancy InN monolayer is also exhibited. It can be seen from Fig. 3(a) that there is a state at the bottom of the conduction band crossing the Fermi level; this indicates that the existence of N-vacancy significantly changes the semiconducting property of the InN monolayer. However, after the adsorption of the NO molecule on the N-vacancy InN monolayer, we can see from the BS in Fig. 3(b) that there is no impurity state crossing the Fermi level. That is, the adsorption of NO molecule is p-doping for the N-vacancy InN monolayer.^25^ On the other hand, two novel states emerge at the top of the valence band, which result from the adsorbed NO molecule based on the molecule DOS of NO shown in Fig. 3(c). From this figure, we can infer that the NO molecule is strongly activated during adsorption: the 1π and 5σ orbitals are shifted to a higher level and spilt into several small states; the spin-down of 2π* orbital becomes occupied even after adsorption and shifts to a level higher than the Fermi level. These findings verify the chemisorption of NO at the N-vacancy site with the N-end position. Moreover, the atomic DOS of the N 2p and In 5p orbitals in Fig. 3(d) exhibits a strong hybridization between the N and the In atoms given the obvious overlaps around −6, −4 and 0 eV, supporting the chemisorption of the NO molecule on the N-vacancy InN monolayer and the formation of stable chemical bonds of In–N.

### 3.3 Repairing processes of the N-vacancy in InN monolayer

**Fig. 4:**
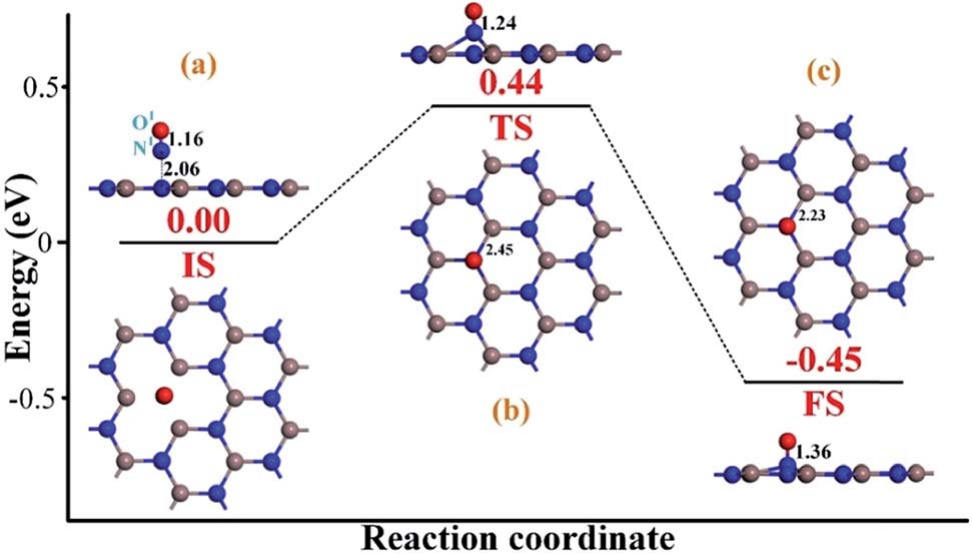
Repairing configurations of the InN monolayer by the NO molecule. (a) IS; (b) TS and (c) FS. The black values are the atomic distances (Å), whereas the red values are the state energies (eV).

To heal the N-vacancy in the InN monolayer, two processes are required, *i.e.* a repairing process and a removal process. The repairing process begins with the physisorption of one NO molecule on the defected InN monolayer, as depicted in Fig. 4(a). The distance between the N^1^ atom and the plane is measured to be 2.06 Å, indicating a large distance of at least 3 Å between the candidate N^1^ and any neighboring In atom. The calculated *E*_ad_ of −0.66 eV and the negative charge of the NO molecule (−0.091*e*) (Table 1) also verify the physisorption nature of the Vac-InN/NO interaction. In Fig. 4(b), we can find that the energy barrier of 0.44 eV must be overcome to reach the transition state (TS) and then fill the N-vacancy using the NO molecule. In the TS, the N^1^–O^1^ bond is elongated to 1.24 Å from 1.16 Å in the initial state (IS), and the N atom is captured by the Vac-InN monolayer with the atom-to-plane distance of 1.20 Å. Moreover, the NO is negatively charged by −0.287*e*, wherein the N^1^ and O^1^ atoms gain 0.074 and 0.122*e* (Table 1) from the Vac-InN monolayer, respectively. In the final state (FS) shown in Fig. 4(c), the atom-to-plane distance becomes further smaller; this indicates that the pioneer NO is stably adsorbed on the Vac-InN monolayer with strong bonds formed between the N^1^ candidate and the neighboring In atoms. The short In–N^1^ bond length of 2.23 Å can confirm this as well, which becomes further shortened when compared with 2.45 Å in TS. For the adsorbed NO molecule, differences occur not only in the N^1^–O^1^ bond length, which elongates up to 1.36 Å, but also in the atomic charges wherein the N^1^ and O^1^ atoms are much more negatively charged by −0.186 and −0.308*e* (Table 1), respectively. Based on the calculated *E*_ad_ of −1.11 eV, chemisorption could be identified for this system. Note that the N^1^–O^1^ bond in NO was weakened to some degree during the repairing process according to the dramatic deformation in its molecular morphology, it is hopeful that the N^1^–O^1^ bond would be workably dissociated by further interacting with another reducing molecule. In this case, the O^1^ atom could be removed, and the Vac-InN monolayer could be repaired.

**Table 1:** Atomic charges of N^1^, O^1^, N^2^ and O^2^ in different states (*e*)

State		N^1^	O^1^	N^2^	O^2^
Repairing process	IS	−0.045	−0.046	—	—
	TS	−0.119	−0.168	—	—
	FS	−0.186	−0.308	—	—
Removing process	IS	−0.189	−0.289	0.023	0.005
	TS	−0.262	−0.308	0.057	−0.034
	FS	−0.373	−0.173	0.075	−0.187

**Fig. 5:**
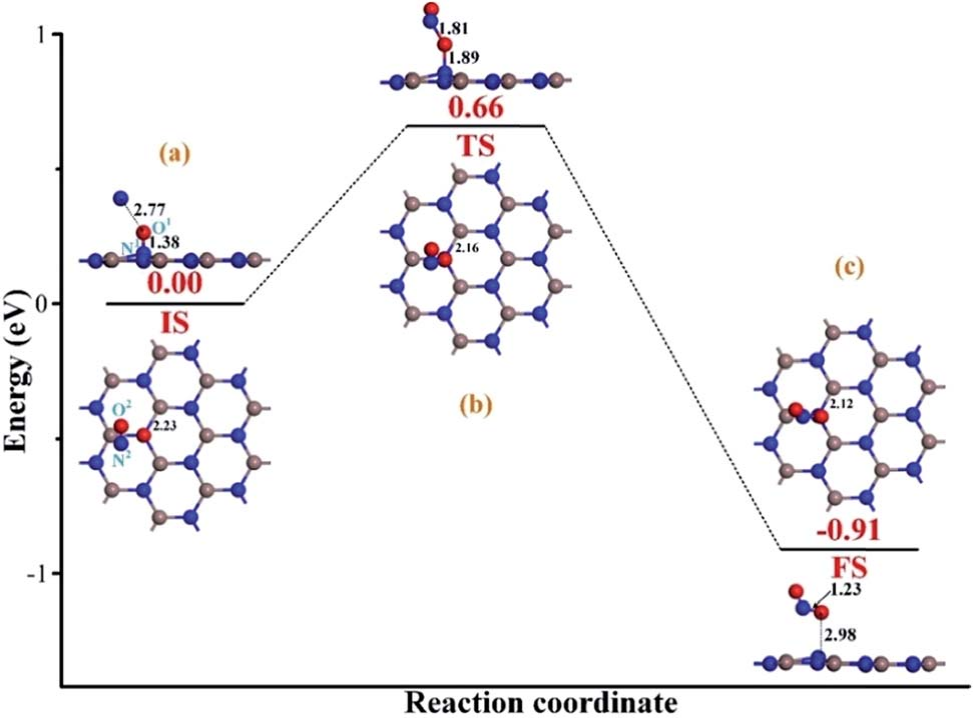
Removing the configuration of extra O atom from the repaired InN monolayer. (a) IS; (b) TS and (c) FS. The black values are the atomic distances (Å); while the red values are the state energies (eV).

After the repairing process, one more NO molecule was introduced as a reducing species to interact with the extra O^1^ atom; this facilitated the dissociation of the N^1^–O^1^ bond in the NO precursor and formation of the separated NO_2_ molecule instead. Then, the repaired InN monolayer could be obtained after the release of the formed NO_2_. Fig. 5(a) demonstrates the initial state (IS) of the removal process. We can see that after interaction, the second NO is located at the left-top above the adsorbed NO, with the distance of 2.77 Å. The N^1^–O^1^ bond length is slightly elongated to 1.38 Å; however, there are no obvious deformations in the structures of the Vac-InN monolayer and the second NO molecule. Moreover, both the N^2^ and the O^2^ atoms are positively charged, donating 0.028*e* to the surroundings, from a molecular point of view; this definitely would lead to electron redistribution in the new system. These findings indicate a weak physisorption for this IS, as further suggested by a small *E*_ad_ of −0.28 eV. With the negatively charged N^1^ and O^1^ atoms (−0.189 and −0.289*e*) as well as positively charged N^2^ atom (0.023*e*) seen in Table 1, there should be electrostatic repulsion between N^1^ and O^1^ and electrostatic attraction between N^2^ and O^1^ at this stage. These forces would enhance the O^1^-removing process significantly. By overcoming the energy barrier of 0.66 eV, the reaction will pass through the TS. As described in Fig. 5(b), the O^1^ atom is trapped by the N^2^ atom with the bond length of 1.81 Å, and the N^1^–O^1^ bond elongates to 1.89 Å, whereas the In–N^1^ bond further shortens to 2.16 Å. These deformations manifest the strong potential for the dissociation of N^1^–O^1^ bond and the formation of a new NO_2_ molecule. Furthermore, electron localization in this TS becomes more evident for the N^1^, O^1^ and N^2^ atoms, which are charged by −0.262, −0.308 and 0.057*e* (Table 1), respectively. These allow the further deformations of the two NO molecules under electrostatic forces towards FS. When to the reaction reaches FS, as portrayed in Fig. 5(c), the formed NO_2_ is desorbed from the repaired InN monolayer, with the N^1^–O^1^ bond length of 2.98 Å. The N^2^–O^1^ bond and In–N^1^ bonds are shortened to 1.23 and 2.12 Å, which are quite close to the lengths of 1.16 and 2.09 Å in the isolated NO and perfect InN monolayer, respectively. The N^1^ atom is negatively charged by −0.373*e* (Table 1), which is a little lower than that of the N-vacancy (−0.421*e*) in the perfect InN monolayer. Upon the release of NO_2_, the N^2^, O^1^, and O^2^ are charged by 0.075, −0.173 and −0.187*e*, respectively. This means that NO_2_ in total accepts 0.279*e* from the repaired InN monolayer, which corroborates with its strong electron-withdrawing capacity when it interacts with certain substrates.^36,37^ In addition, the calculated *E*_ad_ of −1.18 eV in the FS implies a geometrically stable configuration for the NO_2_ removal and InN monolayer reparation.

**Fig. 6:**
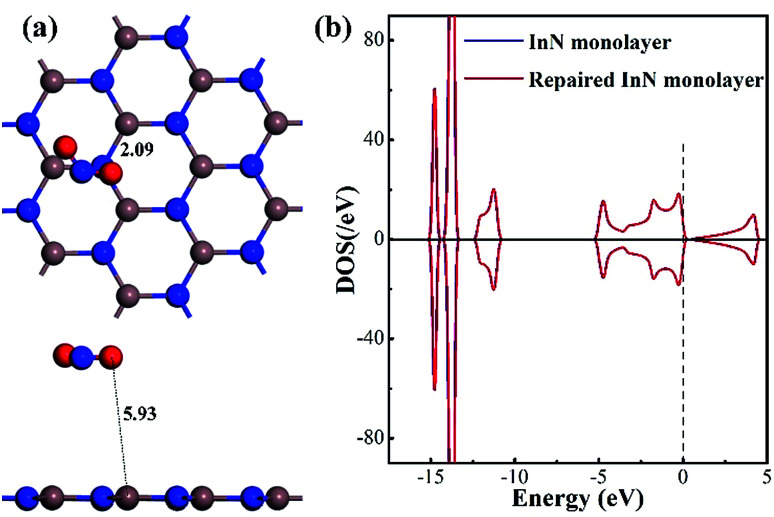
Configuration of the removed NO_2_ from the repaired InN monolayer (a) and DOS of the repaired InN monolayer (b).

As a supplement, we also optimized the configuration wherein the NO_2_ was released from the repaired InN monolayer, keeping a long distance (5.93 Å) with the plane, as exhibited in Fig. 6(a). The In–N^1^ bond recovers to 2.09 Å making the repaired InN monolayer a complete plane and the N^1^ is negatively charged by 0.413*e* indicating the good compatibility of candidate N with the Vac-InN monolayer. At the same time, through the DOS comparison between the intrinsic InN monolayer and repaired counterpart in Fig. 6(b), one can see that the DOS curves of such two systems are completely overlapped at every region, manifesting the recovered electronic behavior for the repaired InN monolayer. In addition, the geometric stability of repaired InN monolayer is further confirmed by the vibrational analysis where the frequency ranging from 113.97 to 1352.98 cm^−1^ is obtained. Based on these results, we presumed that the reparation for Vac-InN monolayer was successfully accomplished.

**Fig. 7:**
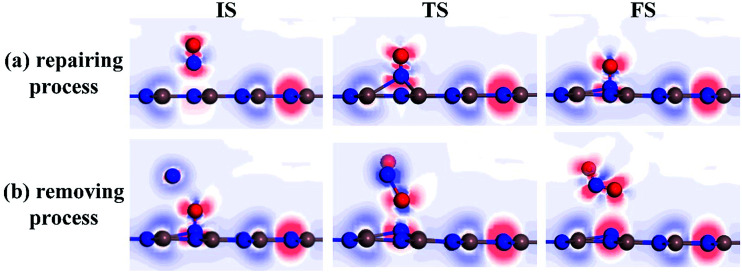
ELF of different states. (a) Repairing process and (b) removing process. The red (blue) region represents charge accumulation (depletion).

To further comprehend the charge-transfer behavior in different states, we implement the electron localization function analysis (ELF), as displayed in Fig. 7. It was found that during the repairing process, the N^1^–O^1^ bond is gradually weakened according to the declined electron accumulation region on the bond, whereas the In–N^1^ bond becomes gradually firmed due to the improved overlaps in electron localizations. Apart from that, the N^1^ and O^1^ atoms maintain negatively charged, which is in accordance with the Hirshfeld method analysis. In the IS of removing process, the N^2^ and O^2^ atoms are slightly positive-charged under the weak physisorption. However, the condition changes remarkably when the reaction reaches the TS, where the electrostatic interactions between N^1^ and O^1^ atoms as well as between N^2^ and O^1^ seem to be visible, given the electron localization distribution. These forces facilitate the dissociation of N^1^–O^1^ bond and formation of novel NO_2_ molecule. In the FS, the N^2^–O^1^ bond becomes further tightened according to the electron accumulation on this bond; while the negatively charged N^1^ atom presents a similar electron accumulation with respect to another native N atom in the InN monolayer. These findings confirmed the feasibility of O^1^-removing by another NO and a good suitability of N^1^ product at the N-vacancy.

Additionally, the energy barrier of 0.44 eV in repairing process could be easily realized at room temperature since a surface reaction at ambient temperature could occur when the energy barrier was smaller than the critical barrier of 0.91 eV.^38–40^ Moreover, the drop energy of 0.45 eV in repairing process could substantially supply for the removal process to overcome the energy barrier of 0.66 eV. Additionally, the strong exothermicity of 0.91 eV in removal process was capable to proceed another repairing–removing cycle to heal any other N-vacancies in InN monolayer. Therefore, we considered that the proposed approach was energetically favorable with good spontaneity.

## 4 Conclusions

In this study, we investigated the reparation of the N-defected InN monolayer by NO molecules; this was theoretically conducted by the first-principles theory. The repairing processes included the adsorption of one NO molecule, filling of the vacancy by the candidate N^1^ and the removal of extra O^1^ by another NO molecule through NO disproportionation. The results obtained herein indicated that the processes were energetically favorable due to the small energy barrier and large energy drop in each reaction. The ELF was also analyzed to further comprehend the charge-transfer behavior in various states. Our study would be meaningful to provide some guidance for the synthesis of a perfect InN monolayer with desirable physicochemical properties, which may also be applied to repair the N-vacancy in other materials.

## Author contributions

Xiaoxing Zhang designed the research, Hao Cui performed the research and wrote this manuscript, while Dachang Chen, Chao Yan and Ying Zhang helped to analyze the data.

## Conflicts of interest

The authors declare no conflict of interest.

## Supplementary Material
